# Genetic Aspects of Conjunctival Melanoma: A Review

**DOI:** 10.3390/genes14091668

**Published:** 2023-08-23

**Authors:** Emily Chang, Hakan Demirci, F. Yesim Demirci

**Affiliations:** 1 Department of Ophthalmology and Visual Sciences, University of Michigan, Ann Arbor, MI 48105, USA; 2 Department of Human Genetics, School of Public Health, University of Pittsburgh, Pittsburgh, PA 15261, USA

**Keywords:** conjunctival melanoma, genetic, molecular, pathways, genes, mutations, structural variations, copy number alterations, UV signature, tumor mutational burden

## Abstract

Conjunctival melanoma (CM) is a rare but aggressive cancer. Over the past decade, molecular studies using rapidly advancing technologies have increasingly improved our understanding of CM genetics. CMs are mainly characterized by dysregulated MAPK and PI3K/AKT/mTOR pathways, driven by commonly mutated (*BRAF*, *NRAS*, *NF1*) or less commonly mutated (*KIT*, *PTEN*) genes. Another group of genes frequently mutated in CMs include *TERT* and *ATRX*, with known roles in telomere maintenance and chromatin remodeling/epigenetic regulation. Uveal melanoma-related genes (*BAP1*, *SF3B1*, *GNAQ/11*) can also be mutated in CMs, albeit infrequently. Additional CM-related mutated genes have increasingly been identified using more comprehensive genetic analyses, awaiting further confirmation in additional/larger studies. As a tumor arising in a partly sun-exposed mucosal tissue, CM exhibits a distinct genomic profile, including the frequent presence of an ultraviolet (UV) signature (and high mutational load) and also the common occurrence of large structural variations (distributed across the genome) in addition to specific gene mutations. The knowledge gained from CM genetic studies to date has led to new therapeutic avenues, including the use of targeted and/or immuno-therapies with promising outcomes in several cases. Accordingly, the implementation of tumor genetic testing into the routine clinical care of CM patients holds promise to further improve and personalize their treatments. Likewise, a growing knowledge of poor prognosis-associated genetic changes in CMs (*NRAS*, *TERT*, and uveal melanoma signature mutations and chromosome 10q deletions) may ultimately guide future strategies for prognostic testing to further improve clinical outcomes (by tailoring surveillance and considering prophylactic treatments in patients with high-risk primary tumors).

## 1. Introduction

Conjunctival melanoma (CM) is a rare malignant neoplasm originating from melanocytes in the basal layer of the conjunctival epithelium [1]. CM accounts for about 5% of all ocular melanomas (about 0.25% of all melanomas) with an annual incidence rate of <1 case per million persons in the Western world [2,3,4,5]. It predominantly occurs in older adults and people of European descent [1,6]; however, people of any descent can be affected by this malignancy. While most published CM data are from North American and European populations, studies from other populations suggest a geographically varying incidence rate, which is likely influenced by both genetic and environmental risk factors [7,8,9]. Unlike uveal melanoma (arising in the uvea—iris, ciliary body, and choroid—of the eye), the incidence of which has remained relatively stable over the last decades, CM is similar to skin melanoma in that its incidence had slowly increased during the past decades (possibly due to an increase in aging population and in ultraviolet (UV) light exposure, which is a known mutagenic factor for sunlight-exposed conjunctiva) [3,5,10,11,12,13,14]. However, more recent epidemiological data do not suggest a continuing rising trend in CM incidence [15,16]. As suggested by comparable tissue size-adjusted incidence figures demonstrated for CM and skin melanoma [9], conjunctiva’s small size/surface appears to be the main reason for the rare occurrence of CM.

**Figure 1:**
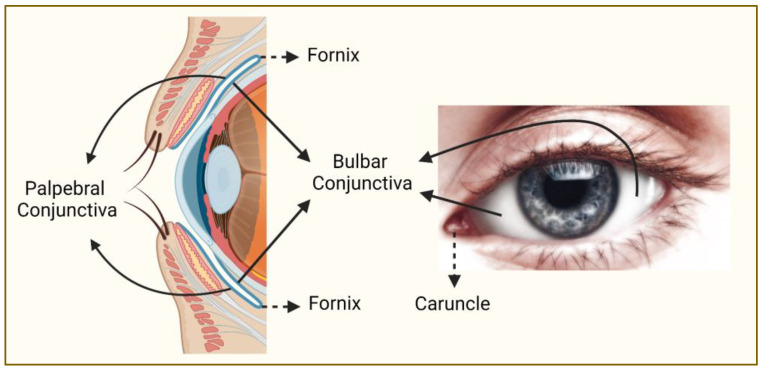
Regions of the conjunctiva affected by melanoma. Conjunctiva (blue line on the left panel) is a partly sun-exposed tissue, and bulbar conjunctiva is more commonly affected by melanoma than non-bulbar (caruncular, forniceal, and palpebral) conjunctiva (this figure was created with BioRender.com and Microsoft PowerPoint).

CMs occur more commonly in sunlight-exposed areas (e.g., bulbar conjunctiva) than in sunlight-protected areas (e.g., behind eyelids) [17,18] (Figure 1). The majority of CMs arise from melanocytic precursor lesions, such as primary acquired melanosis (PAM) with atypia (in up to ~75% of cases) or a pre-existing nevus (in <10% of cases), whereas *de novo* cases account for 15–25% of CMs [17,18,19,20,21]. Localized CM is typically treated with surgical excision and local adjuvant therapy (e.g., cryotherapy, brachytherapy, topical chemotherapy) whereas advanced cases usually require more extensive surgery such as orbital exenteration [1,22]. As suggested by a 10-year local recurrence rate of up to >50% and a 10-year mortality rate of up to >30%, CM is an aggressive cancer highly prone to both local recurrence and metastatic spread (mainly through the lymphatic system) [19,23,24,25,26]. This makes CM not only a potentially sight-threatening condition but also a significantly life-threatening disease, emphasizing the need for a better understanding of its pathogenesis to improve its clinical management. As in any cancer, an increased genetic and molecular understanding of CM development, progression, and spread holds the promise to unravel new prognostic biomarkers and therapeutic targets that can potentially improve the clinical outcomes in CM patients.

**Figure 2:**
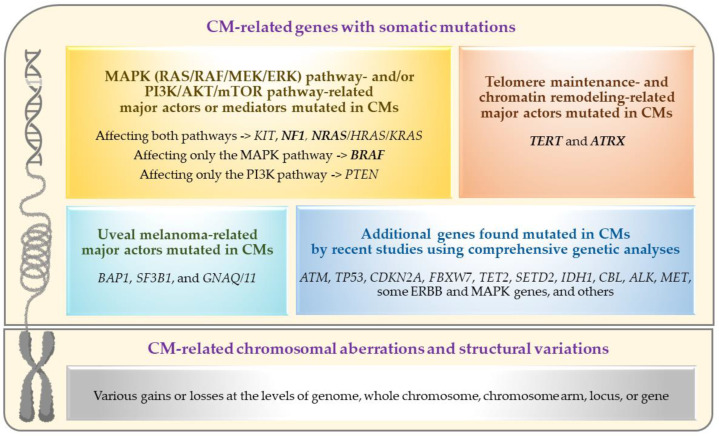
Genetic alterations and affected pathways/genes in primary conjunctival melanomas. Frequently mutated driver genes are marked in **bold** (this figure was created with BioRender.com and Microsoft PowerPoint).

Recent advancements in the genetics/genomics field have significantly accelerated our molecular and biological understanding of various cancers and facilitated the introduction/application of novel revolutionary treatments, such as targeted therapy (for tumors with specific somatic mutations) and immunotherapy (especially for tumors with high somatic mutational load). While CM research has also increasingly benefited from these advancements (as summarized in this review), the genetic aspects of primary CM have yet to be fully understood given the challenges posed by the tumor’s rare occurrence and the paucity of ‘comprehensively analyzed’ large sample collections [27,28,29,30,31,32,33,34]. The primary objective of this review has been to document the current state and understanding of primary CM genetics, including a summary of recent findings worthy of follow-up in future studies. For this purpose, the PubMed search engine was used to identify the articles that contain genetic information on CM, excluding those not published in English. Given the sporadic nature of CM, published genetic studies primarily focused on somatic mutation and/or structural variation analysis of primary tumor samples using targeted or comprehensive analyses. The genetic alterations and affected pathways/genes identified by these analyses are summarized in Figure 2 and further discussed in the following sections.

## 2. Mutations Affecting the Major Actors or Mediators of the MAPK (RAS/RAF/MEK/ERK) and/or PI3K/AKT/mTOR Pathway(s)

The mitogen-activated protein kinase (MAPK) pathway (also known as RAS/RAF/MEK/ERK pathway) and the phosphatidylinositol 3-kinase (PI3K)/AKT/mTOR pathway are the two highly complex and interconnected biological pathways that are often found dysregulated in CMs [9]. These pathways regulate the differentiation, proliferation, and survival of the cells (by transferring growth signals to the nucleus and modulating the expression of multiple genes), and their overactivation (via activation of proto-oncogenes and/or inactivation of tumor suppressor genes) contributes to oncogenesis. As summarized below, mutations found in CMs often affect the major actors or mediators of these two signaling cascades/pathways. A recent in silico structural analysis of CM-associated proteins in these pathways has revealed highly complex protein−protein interactions, which are predicted to be impacted by CM-linked mutations [35].

### 2.1. BRAF (B-Raf Proto-Oncogene, Serine/Threonine Kinase) Mutations

*BRAF* resides at chromosome (chr) 7q34 (https://www.ncbi.nlm.nih.gov/gene/673 (last accessed on 22 July 2023)) and encodes a serine/threonine kinase responsible for activating the downstream MEK, the next kinase in the MAPK (RAS/RAF/MEK/ERK) pathway. Oncogenic *BRAF* mutations cause constitutive activation of the BRAF protein, which in turn leads to constitutive downstream activation of the MAPK pathway (MEK1/2 and ERK1/2) contributing to tumor growth [29,36].

About one third of CMs have been found to harbor *BRAF* mutations [12,27,28,29,30,31,32,33,34,36,37,38,39,40,41,42,43,44]. Nearly all *BRAF* mutations reported to date occur at codon 600 where valine is substituted with glutamic acid (p.V600E, 80–90%) or lysine (p.V600K, 9–20%) or rarely another amino acid; however, mutations affecting other *BRAF* codons have also been detected. These observations are similar to those reported in skin melanomas, whereas *BRAF* mutations do not typically occur in posterior uveal melanomas [44,45,46,47,48,49].

*BRAF* mutations are believed to occur early in CM development, as supported by the observations that they are commonly present in conjunctival nevi and are more frequently found in CMs originating from nevi than PAM [12,36,39,40,42,50,51]. No difference was detected in *BRAF* status of conjunctival nevi in children vs. adults, however, even though children rarely develop CM [42]. Altogether these findings suggest that, while activating *BRAF* mutations are associated with conjunctival melanocytic lesions, additional factors (cumulative molecular events further activating the oncogenic pathways) are necessary for malignant transformation to melanoma.

*BRAF*-mutated CMs appear to arise more often on sun-exposed/bulbar conjunctiva [12,28,39], thus implicating UV radiation as a potential risk factor, although the underlying mechanism remains unknown. In regard to biological variables (sex and age), while some studies reported significant associations with *BRAF*-mutated tumors (more frequently occurring in male [12] and/or relatively younger [12,28,39] patients), other studies did not detect such associations [27,38,40].

Accumulating data do not support the prognostic value of *BRAF* mutations in CM for predicting recurrences, metastases, or survival [8,12,27,28,37]. On the other hand, the therapeutic value of determining the *BRAF* status in CM is increasingly supported by recent studies reporting the beneficial effects of combined BRAF/MEK inhibition in the treatment of recurrent or metastatic CM [52,53,54,55,56] or as neoadjuvant therapy for primary CM [32], and by those recommending its future testing in adjuvant settings [57,58].

### 2.2. NRAS (NRAS Proto-Oncogene, GTPase) Mutations

*NRAS* resides at chr 1p13 (https://www.ncbi.nlm.nih.gov/gene/4893 (last accessed on 22 July 2023)) and belongs to the RAS gene family. It encodes a GTPase located upstream of BRAF in the MAPK (RAS/RAF/MEK/ERK) pathway and can also activate the PI3K/AKT/mTOR pathway [57].

*NRAS* mutations have been identified in about 20% of CMs [27,28,29,30,32,33,34,38,40,59], as similarly observed in skin melanomas, whereas these mutations do not typically occur in posterior uveal melanomas [45,46,47,49]. Commonly detected point mutations at codon 61 (p.Q61R and p.Q61K being the most common) or at codon 12 or 13 (p.G12/13) are believed to favor the GTP-bound active conformation of NRAS protein, leading to an unregulated cellular proliferation [57].

*NRAS* mutations are also commonly found in conjunctival nevi [51]; however, it remains unknown whether there is a link between the *NRAS* status and tumor origin in CM [9]. A recent relatively large CM study [27] identified a link between *NRAS*-mutated tumors and more aggressive behavior (increased risk for metastasis and death), which was also noted as a possibility by another recent study [28]. These recent observations suggest that prophylactic systemic therapy and intensive follow-up may be warranted in patients with *NRAS*-mutated CMs [27]. Although *NRAS*-mutated tumors are suitable targets for the MEK inhibitors (as monotherapy or in combination with PI3K/mTOR inhibitors), data are currently lacking on the therapeutic use of these inhibitors for advanced or metastasized CMs harboring *NRAS* mutations [29,57].

Like in skin melanomas, *NRAS* and *BRAF* mutations are predominantly mutually exclusive in CMs [27,30,32,33,34,40], suggesting that the activating mutations in either of these two key molecules constitute the major oncogenic drivers through the MAPK (RAS/RAF/MEK/ERK) pathway. While *NRAS* is the major RAS gene mutated in CM, the activating mutations in other RAS genes (*HRAS* or *KRAS*) have also been reported in different studies [28,30,38].

### 2.3. NF1 (Neurofibromin 1) Mutations

*NF1* resides at chr 17q11 (https://www.ncbi.nlm.nih.gov/gene/4763 (last accessed on 22 July 2023)) and encodes a tumor suppressor protein that inhibits RAS and thus functions as a negative regulator of both the MAPK and PI3K/AKT/mTOR pathways. Loss-of-function/inactivating *NF1* mutations are therefore associated with increased RAS activity, which in turn leads to overactive MAPK and PI3K/AKT/mTOR signaling.

About one third of CMs have been found to harbor *NF1* mutations [27,28,30,31,32,34,38]. Several different *NF1* mutations have been reported to date, mostly nonsense or frameshift, thus resulting in the loss of function of this tumor suppressor gene/protein [57]. Like in skin melanomas, *NF1* mutations can co-exist with either *NRAS* or *BRAF* mutations in CMs, albeit infrequently [27,28,31,38]. Unlike skin melanomas and CMs, however, uveal melanomas do not typically harbor *NF1* mutations [46,60].

Although the data are currently limited, no association has been reported between *NF1* mutations and clinicopathological features (or prognosis) of CM to date [27,28,38]. Like in skin melanomas, *NF1* mutations appear to occur more frequently in CMs associated with UV signature (typical UV-related C > T or CC > TT nucleotide changes) and higher mutational load [32], suggesting a potential benefit from immunotherapy in patients with *NF1*-mutated tumors [30,38].

### 2.4. KIT (KIT Proto-Oncogene, Receptor Tyrosine Kinase) Mutations

*KIT* resides at chr 4q12 (https://www.ncbi.nlm.nih.gov/gene/3815 (last accessed on 22 July 2023)) and is also known as *c-KIT* because of its initial identification as the cellular homolog of feline sarcoma viral oncogene *v-kit*. *KIT* encodes a receptor tyrosine kinase (RTK), belonging to a large family of transmembrane proteins that are capable of activating several downstream pathways (including MAPK and PI3K/AKT/mTOR pathways) upon stimulation by their ligands [57].

Various oncogenic *KIT* mutations have been found in CMs to date, resulting in constitutive KIT activation and overactive MAPK and PI3K/AKT/mTOR signaling [57]. Like in skin melanomas, *KIT* mutations do not commonly occur in CMs [8,27,28,29,30,38,40,61]; however, *KIT* overexpression is more frequently observed, probably due to other events such as copy number alterations affecting the *KIT* locus [30,40,61].

CMs harboring activating mutations and/or gains of the KIT gene/locus appear to lack both *BRAF* and *NRAS* mutations, suggesting mutual exclusivity between them [28,40,61]. In line with this, *KIT*-mutated CMs are more frequently observed in Asian populations where *BRAF*-mutated CMs occur less frequently [8]. Like *BRAF* and *NRAS* mutations, *KIT* mutations can co-exist with *NF1* mutations in CMs [28,30,57,62], and they can also occur infrequently in posterior uveal melanomas [45,63].

Consistent with the common occurrence of *KIT* mutations in other (sun-protected) mucosal melanomas, a recent study [28] has noted the tendency of these mutations to occur mainly in non-sun-exposed CMs. While no association has been detected between the *KIT* status and survival in CM to date [8,29], *KIT*-mutated tumors are suitable targets for c-KIT inhibitors. It remains unknown, however, whether these inhibitors would be beneficial to CM patients with *KIT*-mutated or *KIT*-amplified tumors [27,28,29,58,62].

### 2.5. PTEN (Phosphatase and Tensin Homolog) Mutations

*PTEN* resides at chr 10q23 (https://www.ncbi.nlm.nih.gov/gene/5728 (last accessed on 22 July 2023)) and encodes a tumor suppressor protein that negatively regulates PI3K and thus functions as an AKT/mTOR pathway inhibitor. Loss of PTEN activity (due to loss-of-function mutation, deletion, or reduced expression of PTEN gene) therefore leads to increased PI3K activity and overactive PI3K/AKT/mTOR signaling.

Like skin melanomas, CMs can demonstrate reduced/lost PTEN expression and upregulation of the mTOR pathway [64]. Uveal melanomas, on the other hand, appear to usually show higher PTEN expression compared to CMs [64]. PTEN’s function is believed to depend on its location (nuclear vs. cytoplasmic), with the nuclear fraction being primarily responsible for tumor suppression [65]. A more pronounced nuclear PTEN loss (weak or no staining for nuclear PTEN) was observed in CMs compared to conjunctival nevi [65], suggesting an important role in oncogenesis/malignant transformation [62].

In addition to the copy number alterations initially found to cause the PTEN loss in CMs [33,40], inactivating *PTEN* mutations have been reported in more recent studies [27,28,29,31]. These recent findings suggest that deleterious *PTEN* mutations can also contribute to the PTEN loss observed in CMs. While *PTEN* mutations appear to be usually mutually exclusive with *NRAS* mutations, they often co-occur with *BRAF* or *KIT* mutations [29].

An association between PTEN loss and CM pigmentation was noted in one previous study [65], which detected a higher nuclear PTEN expression in amelanotic vs. pigmented tumors. While no relation with other CM-related features or prognosis/survival has been reported to date, CMs with PTEN loss could be potential candidates for targeted therapies with mTOR inhibitors [27,62].

## 3. Mutations Affecting the Major Players in Telomere Maintenance and Chromatin Remodeling

In addition to the MAPK and PI3K/AKT/mTOR pathways, telomere maintenance and chromatin remodeling play important roles in CM development and progression as summarized below. In order to gain an unlimited proliferation potential and immortality, cancer cells need telomere maintenance, which can be achieved either by the activation of telomerase reverse transcriptase (TERT) or by the induction of alternative lengthening of telomeres (ALT) pathway (associated with ATRX or DAXX loss) [66]. About 16% of cancers in the Pan-Cancer Analysis of Whole Genomes (PCAWG) dataset were found to harbor somatic mutations in at least one of these three genes (*TERT*, *ATRX*, and *DAXX*) involved in telomere maintenance [66,67]. Growing evidence underscores the importance of telomere maintenance in CM as well, as suggested by the common occurrence of genetic alterations leading to TERT activation [29,30,37,59,68] or loss of ATRX function [27,32,34].

### 3.1. TERT (Telomerase Reverse Transcriptase) Promoter Mutations

*TERT* resides at chr 5p15 (https://www.ncbi.nlm.nih.gov/gene/7015 (last accessed on 22 July 2023)) and encodes the catalytic protein subunit of telomerase (a ribonucleoprotein polymerase), which is responsible for adding short repetitive sequences to the ends of chromosomes to maintain the telomere length. In normal somatic cells, telomeres undergo progressive shortening with successive cell divisions due to repressed telomerase expression, which in turn limits the cells’ replicative capacity, leading to senescence. Abnormal telomerase expression/activity can therefore prevent telomere depletion with successive replications, protecting chromosomes from degradation and making cells ‘immortal’.

Like skin melanomas, CMs frequently harbor *TERT promoter* (*TERTp*) mutations occurring at the same hotspots (in about 35–40% of cases) [29,30,37,59,68]. These mutations can co-occur with *BRAF* or *NRAS* mutations and often display a typical UV signature [29,37]. *TERTp* mutations can cause increased *TERT* expression (via *de novo* transcription factor binding), thus enabling neoplastic cell survival and immortality. It remains to be determined, however, whether other genetic and/or epigenetic mechanisms also contribute to increased TERT expression/activity in CMs [29].

*TERTp* mutations can also be found in PAM lesions (with atypia), but not in conjunctival nevi, implying a role of increased TERT expression/activity in malignant transformation [59,68]. However, more recently, *TERTp* mutations have also been linked to non-PAM-derived CM [29] in addition to PAM with atypia and PAM-derived CM [37,68], suggesting a need for further studies. Unlike CMs, posterior uveal melanomas only rarely harbor *TERTp* mutations [45,68,69].

Recent CM studies [29,37] have demonstrated a link between the presence of *TERTp* mutation and the development of metastasis, indicating its prognostic significance. The presence of *TERTp* mutation may also have therapeutic significance given that the telomerase and reverse transcriptase inhibitors represent future therapeutic options for *TERTp*-mutated tumors [37,68].

### 3.2. ATRX (ATRX Chromatin Remodeler) Mutations

*ATRX* resides at chr Xq21 (https://www.ncbi.nlm.nih.gov/gene/546 (last accessed on 22 July 2023)) and encodes a member of the SWI/SNF family of chromatin remodeling proteins that plays a role in alternative telomere lengthening (through homologous recombination) and in epigenetic regulation (via DNA methylation). Inactivating *ATRX* mutations and loss of ATRX protein are frequently observed in cancers that use the ALT pathway for telomere maintenance, including mucosal melanomas [70,71].

*ATRX* mutations were initially detected in small CM series subjected to comprehensive genetic analyses (identified in ~20–60% of cases) [32,34] and later validated in a relatively large CM series (found in 25% of cases) [27]. Furthermore, the latter study [27] functionally confirmed the loss of ATRX protein expression and ALT positivity in *ATRX*-mutated tumors upon further analysis of a subset of these tumors.

*ATRX* mutations often co-occur with *NF1* mutations and less commonly co-exist with *NRAS* or *BRAF* mutations [27,32,34]. Furthermore, *ATRX*-mutated CMs appear to frequently harbor mutations in genes involved in histone modification and epigenetic regulation (e.g., HDAC and/or SETD genes, *CREBBP*, or *MLLT6*) [32,34]. *ATRX* mutations also appear to co-occur with *TP53* mutations in CMs and other mucosal melanomas [32,71]. By contrast, ATRX loss (via *ATRX* mutations) and TERT activation (via *TERT* amplification or *TERTp* mutations) often show mutual exclusivity in various cancers [71], awaiting a similar confirmation in CM by future studies concurrently analyzing both of these genes for genetic alterations.

The observation of ATRX loss and ALT positivity in both intraepithelial and invasive components of CMs suggests their early occurrence in CM evolution [27]. *ATRX*-mutated CMs appear to arise more commonly in non-sun-exposed areas and show association with less aggressive behavior indicating prognostic significance [27]. *ATRX* mutations may also have therapeutic significance as CMs harboring these mutations would be resistant to anti-telomerase therapies but confer sensitivity to PARP inhibitors [27].

## 4. Mutations Affecting the Genes Typically Involved in Uveal Melanoma Development

Major genetic drivers identified in uveal melanoma include early events activating *GNAQ* (at 9q21) or *GNA11* (at 19p13) and later events involving *EIF1AX* (at Xp22) or *SF3B1* (at 2q33) or *BAP1* (at 3p21) [72,73,74]. Gαq signaling can activate multiple pathways involved in cell growth and proliferation including MAPK, JNK/p38, YAP, mTOR, and β-catenin pathways [72,75]. EIF1AX plays a role in translation initiation (https://www.ncbi.nlm.nih.gov/gene/1964 (last accessed on 22 July 2023)) while SF3B1 is involved in RNA splicing (https://www.ncbi.nlm.nih.gov/gene/23451 (last accessed on 22 July 2023)). BAP1 is a deubiquitinating enzyme (https://www.ncbi.nlm.nih.gov/gene/8314 (last accessed on 22 July 2023)) that functions as a tumor suppressor protein involved in chromatin remodeling, transcription regulation, DNA damage response, and cell death [72,76].

While CM has an overall distinct genetic profile, overlapping more with that of skin and mucosal melanomas than that of uveal melanoma, mutations affecting uveal melanoma-associated genes *BAP1*, *SF3B1*, and *GNAQ/11* have also been detected in CMs, albeit less frequently, including both uveal melanoma-related hotspot mutations and other mutations [27,28,29,31,38,77,78]. In CMs, uveal melanoma-related hotspot mutations appear to often co-occur with the mutations affecting the CM driver genes such as *BRAF*, *NRAS*, *KIT*, *NF1*, and *ATRX* [27,28,29,31].

The presence of uveal melanoma-related hotspot mutations in CMs has been linked to advanced disease and propensity for metastasis and death, indicating an overall poor prognosis similar to that seen in other mucosal melanomas harboring these mutations [27,78,79,80]. Patients with CMs carrying these mutations may therefore benefit from prophylactic treatment and/or more intensive follow-up [27].

## 5. Mutations in Additional Genes Identified by Recent Comprehensive Genetic Analyses

Recent CM studies have identified mutations in additional genes using either unbiased whole genome or exome next-generation sequencing (NGS) or targeted NGS of large panels of cancer-related genes [27,28,30,31,32,34]. Additional genes reported to be mutated in two or more of these CM studies include *ATM* at 11q22 (encoding a cell cycle checkpoint kinase that regulates multiple proteins) [27,28,32], *TP53* at 17p13 (encoding a well-known tumor suppressor protein with diverse functions) [27,28,30,31,32,77], *CDKN2A* at 9p21 (encoding tumor suppressor proteins involved in cell cycle regulation) [27,28,31], *FBXW7* at 4q31 (encoding a tumor-suppressor protein involved in ubiquitin-mediated oncoprotein degradation) [27,32], *TET2* at 4q24 (encoding a methylcytosine dioxygenase involved in epigenetic regulation) [28,34], *SETD2* at 3p21 (encoding a histone methyltransferase involved in epigenetic regulation) [27,32], *IDH1* at 2q34 (encoding an isocitrate dehydrogenase involved in metabolism) [28,30,31], *CBL* at 11q23 (encoding an E3 ubiquitin ligase that interacts with signaling proteins) [28,32,34], *ALK* at 2p23 (encoding a receptor tyrosine kinase) [32,34,77], and *MET* at 7q31 (encoding a receptor tyrosine kinase) [27,28]. Additional mutated genes implicated in a subset of CMs in individual reports (e.g., *CTNNB1*, *ACSS3*, *PREX2*, *APOB*, *RYR1/2*, *SYK*, *NOTCH3*, *CHEK2*, *KMT2A/C*, *ARID2*, *FAT4*, *RB1*, *APC*, as well as some MAPK/MAP2K/MAP3K genes, PIK3CA/B/G genes, additional RTK genes (e.g., *RET* and ERBB genes), and some HDAC and additional SETD genes) [27,28,30,31,32,34,77] warrant further investigation to determine their relevance to CM pathogenesis. While the mutations in the aforementioned genes often co-occur with the mutations in known CM driver genes (e.g., *BRAF*, *NRAS*, *NF1*), those found in tumors lacking these known driver mutations, such as the recently reported mutations in *CTNNB1*, *ARID2*, *TET2*, *TP53*, *RB1*, *RUNX1*, *TSC2*, *CDKN2A*, *CIC*, and some MAPK genes [28,31,32], may be of special interest and worthy of prioritization in follow-up studies.

## 6. UV Light-Related Mutational Signature and Somatic Tumor Mutation Burden (TMB)

Identification of a genomic mutational pattern dominated by C > T substitutions (at dipyrimidine sites), including a subset of CC > TT substitutions, is indicative of UV light-induced DNA damage (known as the ‘UV mutational signature’), which is usually associated with increased tumor mutational burden (TMB) [81].

Tumor genomic profiles (type and frequency of genetic alterations) are known to differ between the two major melanoma groups—epithelium associated (skin, acral, and mucosal) vs. non-epithelium associated (uveal and leptomeningeal) melanomas—as well as among the subtypes within each group [71,82,83]. In contrast to skin melanomas, which are predominantly driven by UV-induced DNA damage (associated with a UV signature and a high TMB), mucosal melanomas occur mostly in sun-protected areas and are often characterized by the absence of UV signature, a lower TMB, but a higher number of structural chromosomal alterations [71,83]. As a tumor originating in a partly sun-exposed mucosal tissue, CM exhibits a distinct genomic profile, which somewhat differs from that of other mucosal melanomas and resembles more that of skin melanoma [82].

Due to UV light-absorbing effects of the cornea and lens, ocular melanomas occurring in areas with different levels of sunlight exposure (CMs vs. anterior uveal (iris) melanomas vs. posterior uveal melanomas) demonstrate corresponding differences in the presence of UV signature and the levels of TMB [58,72,82]. As expected, CMs frequently show a UV signature and higher levels of TMB [13,27,30,32,34,82]. Not all CMs are driven by UV exposure, however, as also supported by a wide range of TMB (mutations per megabase) reported to date [32], but the ones at the higher end of the TMB spectrum constitute the best targets for immunotherapy due to enhanced tumor immunogenicity [84,85,86,87].

Interestingly, both UV-driven and non-UV-driven CMs appear to harbor various large structural variations [32,82], which are commonly seen in mucosal melanomas lacking UV mutational signature [71,83]. It has therefore been proposed that the common tumorigenic process in mucosal melanomas (including CMs) likely involves the accumulation of structural genome variations, and additional processes triggered by UV exposure affect a subset of these tumors [82].

## 7. Chromosomal Aberrations and Structural Variations

In addition to specific gene mutations, various chromosomal aberrations and structural variations (gains or losses at the levels of chromosome/arm, locus, or gene) have also been detected in CMs as summarized below. Copy number alterations (CNAs) have been well studied in uveal melanomas, and some are strongly implicated in prognosis and metastatic outcome, such as loss of chromosome 3 and gain of 8q or 6p [88]. The CNAs in CMs appear to largely differ from those seen in uveal melanomas and follow a pattern similar to that observed in cutaneous and mucosal melanomas.

Numerical chromosomal aberrations such as polyploidy or aneuploidy are observed in CMs, while the structural chromosomal aberrations reported to date include the gains at 1q, 3p, 6p, 7p/q, 8p/q, 11p/q, 12p, 13q, 14p, 17q, and 22q, and the losses at 1p, 3q, 4q, 6q, 8p, 9p/q, 10p/q, 11q, 12q, 15p, 16p/q, 17p, 19p/q, and 21p [30,32,33,34,40,41,89]. The most frequently occurring regional alterations include the amplifications affecting 6p21-25 (especially histone cluster 1 region at 6p22) [30,33,34]. Focal gains involving the major oncogenic drivers of CM (i.e., *BRAF*, *NRAS*, and *TERT*) are observed in a subset of the tumors driven by those [30,40]. Other focal alterations affecting the CM-associated or potentially relevant genes include the amplifications of *KIT* (4q12), *CCND1* (11q13), *CDKN1A* (6p21), *RUNX2* (6p21) and others (e.g., RAF, MAPK, RYR, and BTG genes), and the deletions of *NF1* (17q11), *TP53* (17p13), *CBL* (11q23), *CDKN2A/2B* (9p21), *ASNS* (7q21), and *HLA-A* (6p22) [30,32,33,40,41]. While the putative drivers of recurring CNAs in CM remain largely unknown, accumulating multi-omic data and integrative analyses are expected to provide further information and some answers [72].

Like in skin melanomas, the frequency of CNAs in CMs appears to vary depending on the genetic background and has been shown to be relatively higher in *BRAF/NRAS*-wildtype tumors [33,40]. However, recurring CNAs are observed in all tumor groups, suggesting that CMs share several other pathogenic alterations despite carrying mutually exclusive oncogenic mutations. As for the specific CNAs that differ between the tumor groups, the gains at 1q, 3p, and 17q appear to occur less commonly in *BRAF*-mutated tumors whereas the losses at 10q are found more commonly in this tumor group [33,40]. The tumor suppressor genes affected by the 10q deletions in *BRAF*-mutated tumors include those located at 10q11-23 (*RASSF4*, *C10orf99*, and *PTEN*) and at 10q26 (*DMBT1*, *C10orf90*). While the 10q loss can also be observed in *NRAS*-mutated tumors, its more frequent presence in *BRAF*-mutated tumors supports the hypothesis that these tumors acquire additional event(s) to activate the PI3K-AKT pathway in addition to the MAPK pathway during their evolution [40,62].

While most CNAs identified in CMs to date do not seem to have any clinical/prognostic value, recurrent deletions occurring at 10q24-26 (the region harboring the tumor suppressor genes *NEURL1*, *SUFU*, *PDCD4,* and *C10orf90*) were reported to be associated with increased tumor thickness, lymphatic invasion, and metastatic spread of CM [33].

## 8. Conclusions and Future Directions

Until recently, the genetic aspects of CM had remained elusive. Over the past decade however, molecular studies using targeted or more comprehensive technologies have increasingly advanced our knowledge of DNA mutations, structural variations, and chromosomal aberrations occurring in primary CMs. As a tumor arising in a partly sun-exposed mucosal tissue, CM exhibits a distinct genomic profile, including a frequent observation of UV mutational signature and also a common presence of large structural variations (distributed across the genome) in addition to specific gene mutations. Commonly observed mutations indicate the major roles of dysregulated MAPK (RAS-RAF-MEK-ERK) and PI3K/AKT/mTOR pathways as well as the telomere maintenance and chromatin remodeling/epigenetic regulation mechanisms in CM pathogenesis. While uveal melanoma-related driver genes are infrequently mutated in CMs, an increasing number of other (additional) genes have been implicated by a growing number of CM studies using more comprehensive genetic analyses (awaiting confirmation in additional and larger studies). While our knowledge of CM genetics has significantly improved over the last years, the studies that used high-throughput approaches (i.e., whole exome or genome sequencing) are currently scarce and small in size, thus future studies using these comprehensive approaches in larger cohorts are warranted to uncover CM’s genomic landscape with its full complexity.

Although rare in incidence, CM is a highly recurrent and potentially deadly cancer, and there is currently no consensus on the treatment of locally advanced or metastatic disease. Ongoing advances in our understanding of the genetic and molecular mechanisms involved in CM pathogenesis are therefore important as they increasingly yield new therapeutic targets and options [9,27,57,90,91,92,93,94]. Because of the overlapping genetic features between CMs and skin melanomas (and to a lesser extent between CMs and other mucosal melanomas), targeted therapies tested in those melanomas have also been increasingly applied to individual CM cases with locally advanced or metastatic disease [32,52,53,54,55,56,95,96,97,98], resulting in favorable outcomes in several cases. Immunotherapy may also be beneficial, especially in cases with high TMB, and it has indeed been increasingly investigated as an additional therapy for CM either alone or in combination with targeted therapy [52,95,96,99,100,101,102,103,104,105,106]. In line with these recent developments and therapeutic applications, the implementation of tumor genetic testing into the routine clinical care of CM patients holds promise to further improve and personalize the treatment of this aggressive cancer, thus underscoring the need for larger and longitudinal clinical studies.

Unlike uveal melanomas, little is known about the prognostic implications of genetic changes observed in CMs, therefore predictive genetic testing is not currently standard practice in CM management. However, as tumor genetic testing becomes more accessible and larger CM datasets become available, our knowledge of poor prognosis-related genetic changes has also been advancing (i.e., mutations affecting *NRAS*, *TERT*, or uveal melanoma-related genes/hotspots and recurrent deletions affecting 10q24-26) although awaiting validation in additional/independent studies. This in turn may inform future strategies for prognostic tumor genetic testing to further improve clinical outcomes in CM patients (by tailoring surveillance and considering prophylactic treatments in those with high-risk primary tumors).
